# Geriatric Assessment–Based Stratification to Evaluate Treatment Intensity, Quality of Life, and Survival in Older Patients With Metastatic Colorectal Cancer: Protocol for a Prospective Observational Study

**DOI:** 10.2196/94561

**Published:** 2026-10-09

**Authors:** Takako Hiramatsu, Izumi Okazaki, Kyoko Hosokawa, Kazuhiko Yoshimatsu, Takeshi Nagasaka

**Affiliations:** 1 Department of Nursing Kawasaki Medical School Hospital Kurashiki, Okayama Japan; 2 Department of Digestive Surgery Kawasaki Medical School Kurashiki, Okayama Japan; 3 Department of Advanced Oncology Kawasaki Medical School Kurashiki, Okayama Japan

**Keywords:** metastatic colorectal cancer, older adults, geriatric assessment, frailty, quality of life, patient-reported outcomes, geriatric oncology, prospective observational study

## Abstract

**Background:**

Older patients with metastatic colorectal cancer (mCRC) are a heterogeneous population with wide variability in functional status, frailty, cognition, nutritional reserve, and comorbidity burden. Treatment decisions are often guided primarily by chronological age and performance status, which may not adequately capture vulnerability or patient-centered outcomes in this population.

**Objective:**

The primary aim of this study is to prospectively evaluate the association of baseline geriatric assessment (GA)–derived frailty with overall survival in older patients with mCRC receiving first-line systemic therapy in routine clinical practice; secondary aims are to evaluate the associations of frailty with initial treatment intensity and early changes in quality of life (QOL).

**Methods:**

Baseline GA includes activities of daily living, instrumental activities of daily living, the Clinical Frailty Scale, the Mini-Cog, nutritional assessment, the Charlson Comorbidity Index (excluding cancer-related items), and the Eastern Cooperative Oncology Group (ECOG) performance status. Blood-based prognostic biomarkers, including the prognostic nutritional index and neutrophil-to-lymphocyte ratio, are also collected. Initial treatment intensity is defined according to the first-line systemic regimen selected at baseline, whereas subsequent local therapies are recorded separately. QOL is assessed using the European Organisation for Research and Treatment of Cancer Quality of Life Questionnaire Core 30 (EORTC QLQ-C30) at baseline and 8 to 12 weeks after treatment initiation.

**Results:**

This study was registered in the UMIN Clinical Trials Registry (UMIN000060489) on April 1, 2026. This study received no external funding. Participant recruitment began on April 1, 2026 (the first participant enrolled in June 2026) and is planned to continue for approximately 2.5 years (projected completion: October 2028), with an anticipated sample size of approximately 100 participants; an early QOL follow-up is scheduled for 8 to 12 weeks after treatment initiation for each participant. As of the submission of this revised manuscript (August 2026), 4 participants had been enrolled, baseline QOL assessments had been completed for all 4 participants, and the 8- to 12-week follow-up QOL assessments had been completed for 3 participants; no outcome data were available. Analysis of the primary outcome will be conducted after final survival follow-up, with the main results anticipated for publication in 2030.

**Conclusions:**

This protocol describes a pragmatic GA-based observational framework to examine associations between pretreatment vulnerability, initial treatment intensity, survival outcomes, and patient-reported QOL in older patients with mCRC. Given the exploratory, hypothesis-generating design, the study is expected to generate clinically relevant hypotheses for individualized, patient-centered treatment strategies and future geriatric oncology research.

**Trial Registration:**

UMIN Clinical Trials Registry UMIN000060489; https://tinyurl.com/3mn563er

**International Registered Report Identifier (IRRID):**

DERR1-10.2196/94561

## Introduction

The global population is aging rapidly, leading to an increasing number of older adults diagnosed with metastatic colorectal cancer (mCRC) [1,2]. Because both the incidence and prevalence of colorectal cancer rise steeply with age, the management of older adults with mCRC has become one of the most common challenges in daily oncology practice [1,2]. Advances in systemic therapy have improved survival outcomes, leading to an increasing number of older patients being considered for active treatment [3-6]. However, older adults with mCRC exhibit marked heterogeneity in physical function, frailty, cognitive status, nutritional reserve, and comorbidities, which are not adequately captured by chronological age alone [7-9].

In routine clinical practice, treatment decisions are often based primarily on performance status (PS). Although PS provides a general assessment of functional capacity, it does not fully reflect multidimensional vulnerability in older patients [10,11]. Geriatric assessment (GA), incorporating functional, cognitive, nutritional, and comorbidity domains, has been shown to predict treatment-related toxicity, treatment discontinuation, and survival across various cancer types [12,13]. Moreover, 2 large randomized trials have demonstrated that GA-guided management substantially reduces grade 3 to 5 treatment-related toxicity without compromising survival among older adults initiating systemic therapy [14,15]. Despite growing guideline support for GA [16-18], culminating in the 2023 American Society of Clinical Oncology guideline update that recommends GA with GA-guided management for all patients aged 65 years or older receiving systemic cancer therapy [19], GA is not consistently implemented before treatment initiation in routine oncology practice [16-18]. In older patients with mCRC, the role of GA in informing treatment intensity and quality-of-life (QOL) outcomes remains incompletely defined [8,9].

Older patients with mCRC may receive markedly different first-line regimens, ranging from single-agent fluoropyrimidine to combination chemotherapy, depending on tumor biology, physiological reserve, patient preferences, and physician judgment. However, the association of baseline GA-derived vulnerability with the intensity of initial therapy selected in practice, subsequent survival, and early QOL change remains insufficiently characterized [9,20]. This prospective observational study was designed to address this gap in routine clinical care.

The primary aim of this study is to evaluate the association of baseline GA-derived frailty with overall survival (OS) in patients aged 70 years or older with mCRC; secondary aims are to evaluate the associations of frailty and functional status with (1) the intensity of the initial first-line systemic regimen selected in routine practice and (2) early change in patient-reported QOL. We hypothesize that baseline frailty, defined as a Clinical Frailty Scale (CFS) score of 5 or higher, is associated with lower initial treatment intensity, shorter OS, and greater early deterioration in global health status/QOL, independent of chronological age and Eastern Cooperative Oncology Group (ECOG) PS.

## Methods

### Study Design

This study is a single-center, prospective, noninterventional observational cohort study. No study-specific interventions are mandated, and all treatment decisions are made at the discretion of the treating physicians, in accordance with routine clinical practice.

The study is registered in the UMIN Clinical Trials Registry (UMIN000060489). The current protocol is version 1.0, dated January 27, 2026; any substantive protocol amendments will be submitted to the Ethics Committee for approval and reflected in the trial registry.

Patients and the public were not involved in the design or conduct of this study; the findings are intended to inform patient-centered treatment discussions and future patient-partnered research in geriatric oncology. The overall study flow and assessment timeline are summarized in Figure 1.

**Figure 1:**
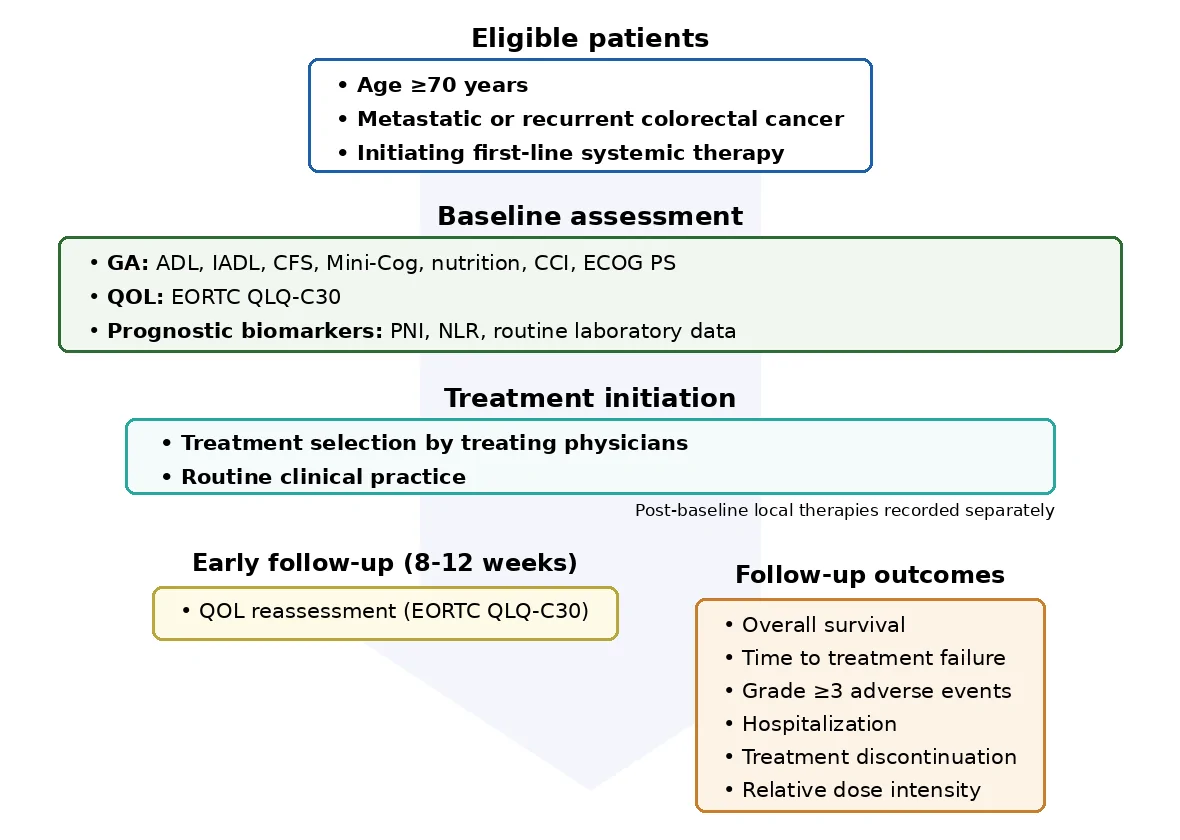
Study flow and assessment timeline. ADL: activities of daily living; CCI: Charlson Comorbidity Index; CFS: Clinical Frailty Scale; ECOG PS: Eastern Cooperative Oncology Group performance status; EORTC QLQ-C30: European Organisation for Research and Treatment of Cancer Quality of Life Questionnaire Core 30; GA: geriatric assessment; IADL: instrumental activities of daily living; NLR: neutrophil-to-lymphocyte ratio; PNI: prognostic nutritional index; QOL: quality of life.

### Study Population

Eligible patients are aged ≥70 years, have histologically confirmed metastatic or recurrent colorectal cancer, have an Eastern Cooperative Oncology Group performance status (ECOG PS) of 0 to 2, and are initiating first-line systemic therapy at Kawasaki Medical School Hospital. Patients with ECOG PS 3 to 4 are not eligible because they are generally not candidates for first-line systemic therapy in routine practice. Patients are screened and enrolled consecutively, and a screening log of all patients assessed for eligibility, including reasons for nonenrollment (ineligibility, lack of capacity to consent, refusal, or other), will be maintained to quantify potential selection at study entry. All participants provide written informed consent prior to enrollment. To preserve real-world applicability, no major disease-based exclusion criteria are prespecified; however, patients receiving best supportive care alone at baseline are not eligible because the study targets individuals starting systemic treatment. Clinical conditions that may transiently alter baseline laboratory biomarkers, such as active infection, will be documented prospectively and addressed in sensitivity analyses.

### Sample Size

On the basis of institutional case volume, approximately 40 to 60 eligible patients are expected to be enrolled annually. Recruitment is planned for approximately 2.5 years, with an anticipated total sample size of about 100 participants. This sample size is feasibility-driven and appropriate for an exploratory prospective cohort study. Although the sample size was determined by feasibility, we estimated the minimally detectable effect sizes for the principal analyses. Assuming a baseline frailty prevalence of approximately 40% and approximately 55 to 65 death events during the study period, the study will have 80% power (2-sided α=.05) to detect a hazard ratio of approximately 2.0 to 2.1 for OS in frail versus nonfrail patients. For the association between frailty and receipt of higher-intensity initial therapy, a sample of 100 participants permits detection of an odds ratio of approximately 3.0 to 3.5 with 80% power, assuming that 50% to 60% of nonfrail patients receive higher-intensity therapy. Associations smaller than these thresholds may not reach statistical significance; accordingly, results will be interpreted with emphasis on effect estimates and CIs rather than statistical significance alone. In particular, the study is not powered to detect hazard ratios smaller than approximately 2.0, and all findings will be reported as exploratory. Because the main analyses are association-based rather than confirmatory, the multivariable models will be prespecified and parsimonious; the primary Cox model is prespecified with 4 parameters (refer to Statistical Analysis), with the number of parameters in all other models restricted according to the number of observed events and the extent of missing data. Emphasis will be placed on effect estimates, CIs, and consistency across sensitivity analyses rather than on formal power-based hypothesis testing alone.

### GA

A structured GA is conducted at baseline prior to treatment initiation and covers 6 domains. Functional status is assessed using the Barthel Index for activities of daily living [21] and the Lawton scale for instrumental activities of daily living (IADL) [22]. Frailty is assessed using the CFS [23,24], cognitive function using the Mini-Cog [25,26], and comorbidity burden using the Charlson Comorbidity Index (CCI) [27,28], excluding cancer-related items. Overall functional capacity is graded using the ECOG PS.

Nutritional status and systemic inflammation are assessed using BMI, weight loss during the 6 months preceding baseline, and blood samples obtained within 14 days prior to the initiation of first-line systemic therapy. This 14-day upper limit reflects the real-world scheduling of outpatient oncology care; at our institution, pretreatment laboratory testing is mandatory before the initiation of systemic chemotherapy and is routinely performed within 7 days of treatment initiation. The actual sampling-to-treatment interval will be recorded for each participant, and sensitivity analyses restricted to samples obtained within 7 days will be performed. All laboratory parameters are measured in the accredited central clinical laboratory of Kawasaki Medical School Hospital as part of routine care, using automated hematology analyzers and standard biochemical assays under routine internal and external quality control; because all biomarkers derive from routine real-time laboratory results, no study-specific sample processing, storage, or batch assays are required. Routine laboratory tests will include complete blood counts with differentials (including total white blood cell, absolute neutrophil, and absolute lymphocyte counts), serum albumin, and alkaline phosphatase to allow for the calculation of established prognostic scores such as the Köhne index [29]. Active infection at the time of blood sampling is operationally defined as clinically documented infection requiring systemic antimicrobial therapy, fever of 38.0 °C or higher, or laboratory evidence of acute inflammation attributed to infection. Patients meeting this definition will not be excluded from enrollment; instead, infection status will be recorded as a covariate, repeat sampling after resolution will be used for biomarker calculation when available, and prespecified sensitivity analyses excluding these patients will be performed. The prognostic nutritional index (PNI) will be calculated based on the method described by Onodera et al [30] using the following formula: PNI = (10 × serum albumin [g/dL]) + (0.005 × total lymphocyte count [/mm^3^]).

The neutrophil-to-lymphocyte ratio (NLR) [31] will be calculated as follows: NLR = absolute neutrophil count/absolute lymphocyte count.

### Frailty Definition

Frailty is primarily defined as a CFS score ≥5. Sensitivity analyses using alternative functional definitions based on the Barthel Index and Lawton IADL scale impairment are planned.

### Comorbidity Assessment

Comorbidity burden is assessed using the CCI, excluding cancer-related items (“any tumor” and “metastatic solid tumor”) to avoid redundancy with tumor burden variables.

To improve reproducibility, comorbid conditions are operationally defined a priori as follows. Mild liver disease (1 point) includes chronic hepatitis or compensated liver disease without clinical signs of decompensation, whereas moderate to severe liver disease (3 points) is defined by documented evidence of decompensation, including ascites, hepatic encephalopathy, variceal bleeding, or Child-Pugh class B or C; when severity cannot be clearly determined, liver disease is conservatively classified as mild. Moderate to severe renal disease (2 points) is defined as chronic kidney disease with an estimated glomerular filtration rate below 30 mL/min/1.73 m², dialysis dependence, renal transplantation, or persistent serum creatinine of 3.0 mg/dL or higher; transient or acute kidney injury is not included. Diabetes without end-organ damage (1 point) is defined as diabetes mellitus without documented complications, whereas diabetes with end-organ damage (2 points) includes documented diabetic nephropathy, retinopathy, neuropathy, or peripheral vascular disease; when both diabetic nephropathy and moderate to severe renal disease are present, points are assigned to both categories.

### QOL Assessment

QOL is assessed using the European Organisation for Research and Treatment of Cancer Quality of Life Questionnaire Core 30 (EORTC QLQ-C30) [32]. Assessments are performed at baseline, prior to treatment initiation, and 8 to 12 weeks after initiation. Key domains analyzed include global health status/QOL, physical functioning, role functioning, fatigue, and appetite loss. Reasons for missing follow-up QOL assessments, including disease progression, hospitalization, treatment discontinuation, or death, will be documented whenever available.

### Schedule of Assessments

The schedule of enrollment, assessments, and follow-up is presented in Table 1.

**Table 1:** Schedule of enrollment, assessments, and follow-up.

Assessment	Baseline (before treatment initiation)	Weeks 8-12 (days 56-84)	Continuous during follow-up
Eligibility screening and informed consent	✓		
Geriatric assessment (Barthel Index, Lawton IADL^a^, CFS^b^, Mini-Cog, CCI^c^, and ECOG PS^d^)	✓		
Laboratory tests (blood counts, albumin, ALP^e^; PNI^f^, NLR^g^, and Köhne index)	✓^h^		
EORTC QLQ-C30^i^	✓	✓	
Treatment exposure and adherence (RDI^j^, dose modifications, and discontinuation)			✓^k^
Adverse events (CTCAE^l^ version 5.0)			✓
Unplanned hospitalization			✓
Survival status and subsequent treatments			✓

^a^IADL: instrumental activities of daily living.

^b^CFS: Clinical Frailty Scale.

^c^CCI: Charlson Comorbidity Index.

^d^ECOG PS: Eastern Cooperative Oncology Group performance status.

^e^ALP: alkaline phosphatase.

^f^PNI: prognostic nutritional index.

^g^NLR: neutrophil-to-lymphocyte ratio.

^h^Within 14 days before treatment initiation.

^i^EORTC QLQ-C30: European Organisation for Research and Treatment of Cancer Quality of Life Questionnaire Core 30.

^j^RDI: relative dose intensity.

^k^During the first 12 weeks and beyond.

^l^CTCAE: Common Terminology Criteria for Adverse Events.

### Treatment Classification

For research purposes, initial treatment intensity will be classified into 2 categories. Higher-intensity initial therapy is defined as combination cytotoxic doublet or triplet chemotherapy with or without a targeted agent (eg, FOLFOX [folinic acid (leucovorin), fluorouracil, and oxaliplatin], CAPOX [capecitabine and oxaliplatin], FOLFIRI [folinic acid (leucovorin), fluorouracil, and irinotecan], or FOLFOXIRI [folinic acid (leucovorin), fluorouracil, oxaliplatin, and irinotecan], with or without bevacizumab or an anti-EGFR [epidermal growth factor receptor] antibody). Lower-intensity initial therapy is defined as fluoropyrimidine monotherapy (eg, infusional 5-fluorouracil/leucovorin or capecitabine) with or without a targeted agent, or immune checkpoint inhibitor monotherapy (eg, pembrolizumab) for microsatellite instability-high/mismatch repair-deficient (MSI-high/dMMR) tumors. The choice of biological agent (bevacizumab versus an anti-EGFR antibody) does not alter the intensity classification. Classification is based on the regimen prescribed at baseline (day 1 of first-line therapy) and is not altered by the delivered relative dose intensity (RDI) or by subsequent modifications; a switch of regimen within the first 8 weeks (eg, owing to toxicity) does not change the baseline classification, and such switches are recorded and described separately. Doublet or triplet regimens prescribed at attenuated starting doses are classified as higher-intensity when the prescribed starting dose is ≥75% of the standard starting dose and as lower-intensity when the prescribed starting dose is <75%. An exhaustive mapping of all first-line regimens anticipated at our institution to the 2 intensity categories is provided in Multimedia Appendix 1.

Receipt of postbaseline local treatment, such as R0 resection, conversion surgery, ablative therapy, or stereotactic body radiotherapy, will be recorded separately as a subsequent treatment variable and will not be used to define baseline treatment-intensity groups. This classification is for research purposes only and does not influence clinical decision-making. In addition, the treating physician’s primary stated reason for the selected regimen (eg, organ function, comorbidity, frailty, or patient preference) will be documented at enrollment to characterize the clinical indications underlying treatment selection.

### Treatment Exposure and Adherence

Because adherence to the initial regimen may confound the relationship between planned treatment intensity and outcomes, treatment delivery will be prospectively captured as follows: (1) RDI, defined as the ratio of delivered to standard planned dose per unit time for each cytotoxic agent during the first 12 weeks of therapy; (2) dose reductions, treatment delays exceeding 7 days, and dose omissions, with documented reasons; (3) early discontinuation within 12 weeks and its reasons (toxicity, disease progression, patient preference, or death); and (4) for oral fluoropyrimidine-containing regimens, adherence assessed from pharmacy dispensing records and patient-reported intake. Analyses relating treatment intensity to outcomes will incorporate RDI as a descriptive and potentially mediating variable, and sensitivity analyses will examine whether frailty-outcome associations are attenuated after accounting for delivered treatment intensity. Criteria for dose modification, interruption, or discontinuation follow institutional standards and the Japanese package inserts and are based on Common Terminology Criteria for Adverse Events (CTCAE)–graded toxicity, disease progression, patient preference, or death; the applied modifications and their reasons will be documented prospectively. All concomitant supportive care, including granulocyte colony-stimulating factor, antiemetics, and specialist palliative care, is permitted, and none is prohibited; postbaseline local therapies are recorded separately as described in the Treatment Classification section.

### Outcomes

The primary outcome is OS, defined as the time from initiation of first-line systemic therapy to death from any cause. The primary associational question of this study is whether baseline frailty (CFS ≥5) is associated with OS; the associations of frailty with initial treatment intensity and with early QOL change are prespecified secondary questions.

Secondary outcomes are defined as follows. Time to treatment failure is the time from initiation of first-line therapy to discontinuation of the initial regimen for any reason (disease progression, toxicity, patient preference, switch of regimen, or death); patients remaining on the initial regimen are censored at the date of last follow-up. Grade ≥3 adverse events are graded according to the National Cancer Institute CTCAE (version 5.0) and recorded systematically at routine clinical visits. Hospitalization is restricted to unplanned admissions and summarized as the proportion of patients admitted, the admission rate per person-year, and the cumulative length of stay in days. RDI is the delivered dose intensity divided by the standard planned dose intensity for each cytotoxic agent over the first 12 weeks of therapy, with omitted doses counted as zero delivered dose. Change in QOL is the change in EORTC QLQ-C30 scores between baseline and the 8- to 12-week assessment (56-84 days after treatment initiation), with an absolute change of ≥10 points regarded as clinically meaningful [33]. The treatment discontinuation rate is also recorded. Receipt of postbaseline local treatment will be documented as an exploratory clinical outcome.

### Estimands

For the primary question, the population comprises patients aged 70 years or older with mCRC and ECOG PS 0 to 2 initiating first-line systemic therapy; the exposure is baseline frailty (CFS ≥5) compared with nonfrailty (CFS <5); the outcome is OS; time 0 is the initiation of first-line systemic therapy; and the summary measure is the hazard ratio adjusted for age, primary tumor laterality, and *RAS/BRAF* status; initial treatment intensity is not adjusted for because it is a potential mediator of the frailty-survival association. For the secondary treatment-intensity question, the same population and exposure contrast apply; the outcome is receipt of higher-intensity versus lower-intensity initial therapy determined at baseline; time 0 is the time of treatment selection at enrollment; and the summary measure is the covariate-adjusted odds ratio. For the secondary QOL question, the outcome is the change in EORTC QLQ-C30 scores from baseline to 56 to 84 days after treatment initiation; time 0 is the initiation of first-line therapy; and the summary measure is the adjusted difference in mean score change between frail and nonfrail patients, defined as a while-alive estimand among participants alive at the follow-up assessment; deaths before the 8- to 12-week assessment are reported separately and are not imputed. All estimands are associational; no causal treatment-effect estimand is targeted.

### Mitigation of Confounding by Indication

Because treatment intensity is selected by treating physicians rather than by randomization, confounding by indication is an inherent risk of this design. The following prespecified strategies will be applied to mitigate and characterize this bias: (1) multivariable adjustment for the principal clinical drivers of regimen selection, namely age, ECOG PS, frailty status, primary tumor laterality, and molecular characteristics; (2) exploratory descriptive examination of frailty-outcome associations stratified by initial treatment intensity, with the unstratified 4-parameter model constituting the primary analysis; (3) exploratory sensitivity analyses using propensity scores for receipt of higher-intensity therapy, implemented as inverse probability of treatment weighting, with multivariable adjustment constituting the primary analytic approach; the propensity model will be limited to 3 to 4 covariates, and the weight distribution (including mean and maximum weights) and standardized mean differences before and after weighting will be reported, with weight truncation at the 1st and 99th percentiles; these analyses are exploratory; (4) prospective documentation of the primary stated reason for regimen selection, enabling descriptive characterization of treatment indications; and (5) explicit interpretation of all treatment-intensity analyses as associational rather than causal, using the target-trial framework as a conceptual guide only [34]. All findings regarding frailty, treatment intensity, and survival will be interpreted strictly as observational and noncausal.

### Statistical Analysis

Baseline characteristics will be summarized descriptively. The primary analysis will examine the association between baseline frailty status and OS. OS will be estimated using Kaplan-Meier methods and analyzed using Cox proportional hazards regression. Multivariable models will be prespecified and parsimonious. The primary Cox model for the frailty-OS association is prespecified to contain exactly 4 parameters: frailty status (CFS ≥5), age, primary tumor laterality, and molecular characteristics (*RAS/BRAF* status). Initial treatment intensity is deliberately excluded from this primary model because it lies on the potential causal pathway from frailty to survival and may act as a mediator; adjusting for a mediator would bias the total association of interest. ECOG PS is likewise not included in the primary model because the CFS and ECOG PS capture partially overlapping constructs. With the anticipated 55 to 65 death events, this 4-parameter specification provides approximately 14 to 16 events per parameter, exceeding the commonly applied guidance of 10 events per parameter; Firth penalized likelihood or comparable penalized approaches will be applied if event counts are lower than anticipated. Two prespecified sensitivity and exploratory analyses will assess robustness. First, a sensitivity model additionally including ECOG PS is prespecified to assess the stability of the frailty-OS association, and pairwise Spearman correlations and variance inflation factors among the CFS, ECOG PS, Barthel Index, Lawton IADL, and CCI will be reported. Second, an exploratory mediation analysis will add initial treatment intensity to the model using appropriate mediation methods; the frailty coefficient from this model will be interpreted strictly as the direct effect of frailty not mediated through treatment intensity, and not as the total association. The association between frailty and initial treatment intensity itself will be analyzed as a separate secondary outcome using logistic regression, as described below.

Baseline PNI and NLR will be analyzed primarily as continuous variables. Potential nonlinear associations with OS will be explored using restricted cubic splines when data permit. For secondary and sensitivity analyses, these biomarkers will also be categorized using pragmatic, prespecified thresholds (NLR 2.0 and PNI 45).

The association between baseline GA-derived frailty and receipt of higher-intensity initial therapy will be examined using logistic regression. QOL trajectories between baseline and 8 to 12 weeks will be analyzed using linear mixed-effects models, with time, frailty status, and initial treatment intensity as fixed effects; clinically interpretable deterioration in key EORTC QLQ-C30 domains will also be summarized descriptively. Missing baseline covariates will be handled using multiple imputation by chained equations with 20 imputations; the imputation model will include the exposure (frailty status), all analysis-model covariates (age, ECOG PS, primary tumor laterality, and *RAS/BRAF* status), the outcome information (event indicator and cumulative baseline hazard), and auxiliary variables (PNI, NLR, and the functional measures). Complete-case analyses will be performed as sensitivity analyses. Because missing follow-up QOL data may be related to disease progression or death, the QOL change analysis is defined as a while-alive estimand: deaths before the scheduled reassessment will be reported separately and will not be imputed in the linear mixed-effects model. Remaining missingness among survivors will be regarded as potentially missing not at random: reasons for missingness will be documented, and delta-based pattern-mixture sensitivity analyses will be performed in addition to analyses assuming missingness at random to evaluate the robustness of QOL findings. Receipt of postbaseline local treatment will be described separately and, if analyzed in relation to survival, will be handled using time-dependent or landmark approaches. Sensitivity analyses will include alternative frailty definitions and exclusion of patients with MSI-high/dMMR tumors. Given the exploratory nature of the study, emphasis will be placed on effect sizes, CIs, and consistency of findings across sensitivity analyses rather than on hypothesis testing alone.

### Ethical Considerations

This study was approved by the Ethics Committee of Kawasaki Medical School (approval 7048-00) in February 2026. Written informed consent will be obtained from all participants prior to enrollment.

Because patients with cognitive impairment are intentionally not excluded, the consent process includes an assessment of decision-making capacity. Capacity is evaluated clinically by the attending physician at enrollment, based on the patient’s ability to understand, retain, and weigh the study information and to communicate a choice; the Mini-Cog is used as a cognitive screening aid but not as the determinant of capacity. Patients judged to lack the capacity to consent will not be enrolled. Consent materials are written in plain language, and family members are encouraged to attend consent discussions.

All study data will be pseudonymized using a study identification number; the linkage key will be stored separately on a password-protected institutional server accessible only to authorized investigators, and no identifiable data will leave the institution. Study data are entered into a structured electronic database with predefined variable coding, input validation, and automated range checks to promote data quality. Given the noninterventional design, no independent data monitoring committee is convened; study conduct and data quality are overseen by the principal investigator, with periodic internal review of data completeness and consistency. Results will be disseminated through peer-reviewed publication and presentation at academic conferences irrespective of the direction of the findings, and authorship will follow the criteria of the International Committee of Medical Journal Editors.

## Results

This study was registered in the UMIN Clinical Trials Registry (UMIN000060489) on April 1, 2026. Recruitment began on April 1, 2026, and is planned to continue for approximately 2.5 years (projected completion: October 2028), with an anticipated sample size of about 100 participants.

Baseline GA and QOL evaluations are performed prior to the initiation of first-line systemic therapy. Follow-up assessments, including a QOL evaluation 8 to 12 weeks after treatment initiation, are conducted in accordance with the study protocol.

As of the submission of this revised manuscript (August 2026), 4 participants had been enrolled. Although recruitment formally began on April 1, 2026, enrollment of the first participant was delayed until June 2026 owing to the administrative setup of the study systems; the current accrual therefore reflects the early start-up phase, and accrual is expected to increase as institutional referral pathways are consolidated. Baseline (before treatment) QOL assessments had been completed in all 4 enrolled participants, and the 8- to 12-week follow-up QOL assessment had been completed in 3. Data collection is in progress, and no outcome data were available. Analysis of the primary outcome will be conducted after final survival follow-up, and the main results are anticipated for publication in 2030.

## Discussion

This prospective observational protocol is designed to examine the association between baseline GA-derived vulnerability and initial treatment intensity, survival outcomes, and early patient-reported QOL in older patients with mCRC. The methodological framework was intentionally developed to improve the clinical interpretability of geriatric oncology data while preserving the pragmatic character of routine clinical practice.

A central feature of the protocol is its noninterventional design. Treatment selection is determined by treating physicians based on routine clinical judgment and patient preference, allowing the study to capture naturally occurring practice variation among older adults. At the same time, this design does not eliminate confounding by indication or other sources of bias inherent to observational research [35]. Accordingly, all findings regarding frailty, treatment intensity, and survival will be interpreted strictly as observational, noncausal estimates of association rather than as causal treatment effects [34], and the prespecified mitigation strategies described in the Methods section, including multivariable adjustment, propensity score–based sensitivity analyses, and documentation of the stated reasons for regimen selection, will be applied throughout.

Another strength is the structured operationalization of GA domains. Functional status, frailty, cognition, nutritional reserve, and comorbidity burden are assessed using validated instruments with explicit scoring rules. Cancer-related items are excluded from the CCI to reduce redundancy with tumor burden variables, and a priori definitions are specified for renal disease, liver disease, and diabetes with end-organ damage to improve reproducibility.

The temporal design is also deliberate. Baseline GA captures pretreatment vulnerability, whereas QOL reassessment at 8 to 12 weeks was pragmatically selected to capture early treatment burden, and interpretation of change scores will be informed by published work on clinically meaningful change in EORTC QLQ-C30 scores [33]. This design may be particularly relevant in older adults, because early changes in symptoms or function may be clinically informative when interpreting the subsequent treatment course.

From an analytic perspective, several decisions were made to strengthen internal consistency. Initial treatment intensity is defined by the first-line systemic regimen chosen at baseline, whereas postbaseline local therapies, such as conversion surgery, resection, ablation, or radiotherapy, are recorded separately. This separation is important because subsequent local therapy is partly determined by postbaseline response and survival time and therefore should not be used to define baseline exposure or groups [36,37]. Biomarkers such as PNI and NLR will be modeled primarily as continuous variables, with prespecified thresholds reserved for secondary and sensitivity analyses. Because initial treatment intensity is a potential mediator of the frailty-survival association, it is excluded from the primary Cox model and examined only in an exploratory mediation analysis interpreted as a direct effect. In addition, prospective capture of RDI, dose modifications, and early discontinuation will allow delivered, rather than merely planned, treatment intensity to be characterized and incorporated into sensitivity analyses.

The protocol also prespecifies approaches to missing data and limited sample size. Multivariable models will be kept parsimonious and focused on clinically essential covariates. Missing baseline covariates will be addressed using multiple imputation by chained equations, and the handling of incomplete follow-up QOL data will explicitly consider the possibility that missingness may be related to progression, hospitalization, or death [38,39]. Sensitivity analyses using alternative frailty definitions and exclusion of MSI-high/dMMR tumors are planned to assess robustness.

The decision to apply minimal exclusion criteria enhances generalizability. Older patients with cognitive impairment, functional dependence, or multiple comorbidities—groups frequently underrepresented in clinical trials—are intentionally included whenever feasible [8,40]. Nevertheless, the single-center design carries specific limitations that warrant explicit consideration. First, selection bias may arise from local referral patterns: as a university hospital, our institution may preferentially receive patients referred for specialist management, whereas older patients managed with best supportive care in the community without referral are not captured. Second, practice variation is inherently constrained, because regimen selection reflects the preferences of a limited number of treating physicians and institutional care pathways, which may differ systematically from those in community practice or other health systems. Third, the modest sample size limits the precision of subgroup estimates. Fourth, although cognitive impairment itself is not an exclusion criterion, patients judged to lack the capacity to consent are not enrolled, which limits generalizability to the most severely frail or cognitively impaired individuals; the consecutive screening log will be used to quantify this selection at study entry. For these reasons, the estimated associations may not generalize directly to other settings. To support the assessment of external validity, detailed baseline characteristics will be reported and compared with published population-based cohorts, and the explicit operational definitions of GA domains and treatment intensity in this protocol are intended to enable harmonized replication. We plan to use this cohort as the foundation for a future multicenter GA-informed study, and external validation of any observed prognostic associations in independent cohorts will be required before clinical application.

In summary, this protocol integrates pragmatic prospective data collection, structured GA, objective laboratory biomarkers, and patient-reported outcomes to address an important gap in geriatric oncology [41]. By focusing on associations among pretreatment vulnerability, initial treatment intensity, survival, and early QOL, the study aims to generate clinically actionable hypotheses and support the development of future multicenter GA-informed research [12,42]. Consistent with its exploratory design, findings will be reported as hypothesis-generating, and nonsignificant or borderline associations will not be overinterpreted.

## Data Availability

The datasets generated and/or analyzed during the current study will be available from the corresponding author on reasonable request after publication of the main results.

## Appendix

Multimedia Appendix 1 Exhaustive mapping of anticipated first-line systemic regimens to treatment-intensity categories.

## Abbreviations

CAPOX: capecitabine and oxaliplatin
CCI: Charlson Comorbidity Index
CFS: Clinical Frailty Scale
CTCAE: Common Terminology Criteria for Adverse Events
ECOG: Eastern Cooperative Oncology Group
EGFR: epidermal growth factor receptor
EORTC QLQ-C30: European Organisation for Research and Treatment of Cancer Quality of Life Questionnaire Core 30
FOLFIRI: folinic acid (leucovorin), fluorouracil, and irinotecan
FOLFOX: folinic acid (leucovorin), fluorouracil, and oxaliplatin
FOLFOXIRI: folinic acid (leucovorin), fluorouracil, oxaliplatin, and irinotecan
GA: geriatric assessment
IADL: instrumental activities of daily living
mCRC: metastatic colorectal cancer
MSI-high/dMMR: microsatellite instability-high/mismatch repair-deficient
NLR: neutrophil-to-lymphocyte ratio
OS: overall survival
PNI: prognostic nutritional index
PS: performance status
QOL: quality of life
RDI: relative dose intensity
