# Supplementary material for: Geriatric Assessment–Based Stratification to Evaluate Treatment Intensity, Quality of Life, and Survival in Older Patients With Metastatic Colorectal Cancer: Protocol for a Prospective Observational Study
Source: JMIR Res Protoc. 2026 Oct 9;15:e94561. doi: 10.2196/94561 (PMC13694998; doi:10.2196/94561)
Supplement: Supplementary file 1 [file resprot_v15i1e94561_app1.docx]

**Multimedia Appendix 1. Exhaustive mapping of anticipated first-line systemic regimens to treatment-intensity categories.**

Geriatric Assessment–Based Stratification to Evaluate Treatment Intensity, Quality of Life, and Survival in Older Patients with Metastatic Colorectal Cancer: Protocol for a Prospective Observational Study (ms#94561)

| First-line regimen prescribed at baseline (day 1) | Biologic agent permitted | Intensity classification |
| --- | --- | --- |
| mFOLFOX6 (oxaliplatin + infusional 5-FU/leucovorin) | None, bevacizumab, or anti-EGFR antibody (cetuximab/panitumumab)^a^ | Higher |
| CAPOX (oxaliplatin + capecitabine) | None or bevacizumab | Higher |
| FOLFIRI (irinotecan + infusional 5-FU/leucovorin) | None, bevacizumab, or anti-EGFR antibody (cetuximab/panitumumab)^a^ | Higher |
| FOLFOXIRI (oxaliplatin + irinotecan + infusional 5-FU/leucovorin) | None or bevacizumab | Higher |
| Doublet or triplet prescribed at an attenuated starting dose ≥75% of standard^b^ | As above | Higher |
| Doublet or triplet prescribed at an attenuated starting dose <75% of standard^b^ | As above | Lower |
| Infusional 5-FU/leucovorin monotherapy (simplified de Gramont) | None or bevacizumab | Lower |
| Capecitabine monotherapy | None or bevacizumab | Lower |
| Pembrolizumab monotherapy (MSI-high/dMMR tumors only) | Not applicable | Lower |

^a^ Anti-EGFR antibodies are restricted to *RAS/BRAF* wild-type, principally left-sided tumors, per standard practice. The choice of biologic agent (bevacizumab versus an anti-EGFR antibody), or the omission of a biologic agent, does not alter the intensity classification.

^b^ The percentage refers to the prescribed starting dose of the cytotoxic backbone relative to the standard starting dose. Classification is based on the regimen prescribed at baseline (day 1 of first-line therapy) and is not altered by the delivered relative dose intensity or by subsequent dose modifications. A switch of regimen within the first 8 weeks (eg, owing to toxicity) does not change the baseline classification; such switches are recorded and described separately.

Regimens not listed above are not anticipated at our institution for first-line therapy in this population. Should an unlisted regimen be prescribed, it will be classified by the study team according to the same principles (combination cytotoxic therapy = higher; single-agent fluoropyrimidine or immune checkpoint inhibitor monotherapy = lower) before database lock, with the decision documented.
